# Revalidation to single ventricle pathway with single ventricular assist device: Proof of concept

**DOI:** 10.1016/j.xjtc.2024.02.021

**Published:** 2024-03-07

**Authors:** Eiri Kisamori, Manan Desai, Jennifer H. Lindsey, Shriprasad R. Deshpande, Gil Wernovsky, Yves d’Udekem

**Affiliations:** a Division of Cardiac Surgery, Children’s National Hospital, The George Washington University, Washington, DC; b INOVA Children’s Cardiology, Fairfax, VA; c Division of Cardiology, Children’s National Hospital, The George Washington University, Washington, DC

**Figure:**
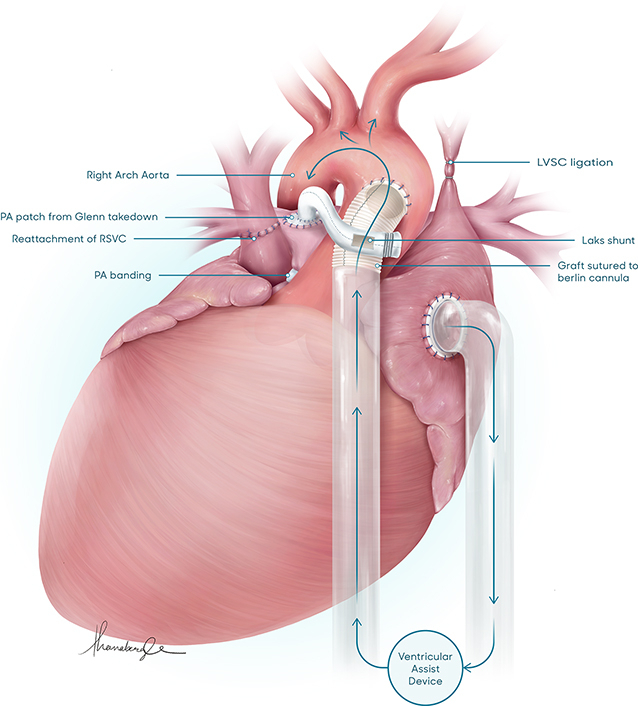
Ventricular assist device and shunt in a patient with a single ventricle.

**Central Message**

Ventricular assist device implantation improved pulmonary vascular resistance, ventricular function, and atrioventricular valve regurgitation in a patient with a single ventricle.

The association of obstructed total anomalous pulmonary venous drainage (TAPVD) and a single-ventricle condition carries a guarded prognosis,^1^^,^^2^ with a mortality of up to 90% within the first year of life.^2^ In recent times, long-term circulatory support of single-ventricle conditions has found success as a bridge to transplant, but not as a bridge to recovery. We present a case of heterotaxy syndrome, unbalanced atrioventricular septal defect (AVSD), severe common atrioventricular valve regurgitation, reduced ventricular function, and TAPVD in whom ventricular assist device (VAD) implantation resulted in a gradual improvement in ventricular function, AV valve regurgitation, and pulmonary vascular resistance leading to the successful weaning of the support and resumption of the single-ventricle pathway rather than heart transplant.

## Clinical Summary

**Figure 1:**
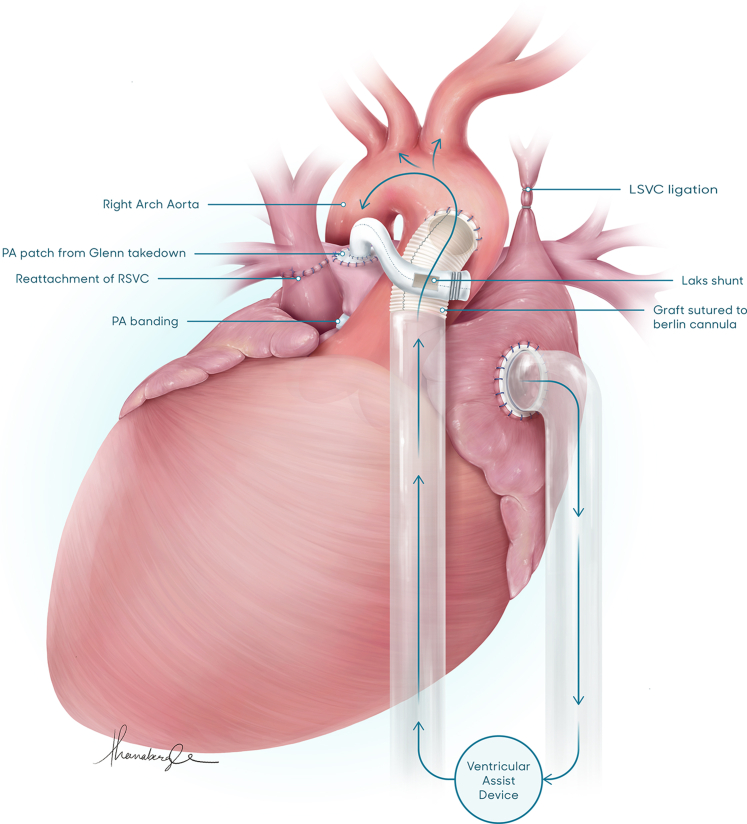
Bidirectional cavopulmonary shunt (*BCPS*) takedown and ventricular assist device (*VAD*) implantation: The BCPS was taken down, and a 6-mm atrial cannula was secured into the right atrium. An 8-mm polytetrafluoroethylene (*PTFE*) graft, secured to a 5/6 left ventricle apical vent cannula, was used as an outflow cannula, and was sutured to an opening made in the ascending aorta. A 4-mm PTFE graft was used to create a systemic to pulmonary shunt using the Laks technique. PediMag (Abbott) VAD support was initiated. *LSVC*, Left superior vena cava; *PA*, pulmonary artery; *RVSC*, right superior vena cava.

This male newborn infant was diagnosed with heterotaxy syndrome, dextrocardia, unbalanced AVSD, hypoplastic right ventricle, moderate common AV valve regurgitation, and obstructed TAPVD to the left superior vena cava (Video 1). Institutional review board approval and informed consent were waved due to the nature of this study. Due to poor prognosis associated with the constellation of findings, the parents decided to opt for compassionate care. The patient survived and at 3 months, goals of care were discussed again. He underwent placement of a bidirectional cavopulmonary shunt (BCPS), a TAPVD repair, and pulmonary artery banding to preserve some antegrade flow. Two days after the surgery, the patient was extubated. On postoperative day 3, he experienced cardiac arrest in a cardiac intensive care unit and was placed on extracorporeal membrane oxygenation during resuscitation. He was weaned off extracorporeal membrane oxygenation on postoperative day 8, but experienced repeated desaturations and dependency on mechanical ventilation. At this stage, the takedown of the BCPS to a systemic to pulmonary artery shunt was deemed necessary but was a high risk procedure due to ventricular dysfunction, severe AV valve regurgitation, and labile pulmonary vascular resistance. On postoperative day 11, it was decided to proceed with the elective addition of a VAD. At 5.3 kg, the BCPS was taken down, and a 6-mm atrial cannula was secured into the right atrium. An 8-mm polytetrafluoroethylene (PTFE) graft, secured to a 5/6 left ventricle apical vent cannula, was sutured to the ascending aorta. A 4-mm PTFE graft was used to create a systemic to pulmonary shunt using the Laks technique with the proximal end on the outflow graft. PediMag (Abbott) centrifugal VAD support was initiated (Figure 1). The main pulmonary artery was divided. The AV valve repair could not be performed because of the patient’s dextrocardia anatomy. Over time, the patient's ventricular function normalized and common AV valve regurgitation improved significantly to the mild range. At age 6 months, 8 weeks after VAD implantation and BCPS takedown, the VAD was explanted with cardiopulmonary bypass. The 8-mm PTFE graft was clamped and divided between the origin of the Laks shunt and the outflow cannula, leaving a small segment of the 8-mm PTFE graft from which the 4-mm Laks shunt was attached. The patient recovered well from this operation with normal ventricular function and mild AV regurgitation at discharge. The patient returned at age 14 months for an elective BCPS and did well with no postoperative complications. At age 2 years, he is thriving while awaiting Fontan completion.

## Discussion

We hereby report successful VAD support of a patient with heterotaxy, unbalanced AVSD, obstructed TAPVD, AV regurgitation, and poor ventricular function as a bridge to recovery after failure of BCPS circulation. Despite the overall growth and progress made in VAD support of patients with single ventricles, outcomes reported with this strategy have been suboptimal. A previous study demonstrated high mortality in stage 1 and stage 2 patients compared with stage 3 patients; 6-month survival was 30% for stage 1, 40% for stage 2, and 95% for stage 3.^3^ Bleiweis and colleagues^4^ reported better outcomes with various constructions of single-ventricle support, which inspired us to use a Y branching of the outflow graft cannula in our case. They performed VAD insertion in 15 patients with functionally single ventricle.^4^ Ten patients (67%) survived and achieved heart transplant, and 5 patients died (33%).^4^

Sustained improvement of AV valve regurgitation with VAD support has been described in adults with mitral valve regurgitation but not in patients with a single ventricle.^5^ We believe that the observed improvement in ventricular function and AV valve regurgitation following VAD implantation in this patient is a proof of the concept that patients with failing single-ventricle circulation can be bridged to recovery by temporary VAD support. We believe that this concept may open new avenues of treatment.

## Conflict of Interest Statement

The authors reported no conflicts of interest.

The *Journal* policy requires editors and reviewers to disclose conflicts of interest and to decline handling manuscripts for which they may have a conflict of interest. The editors and reviewers of this article have no conflicts of interest.

## Supplementary Data

Video 1 Clinical summary of the patient. Video available at: https://www.jtcvs.org/article/S2666-2507(24)00077-4/fulltext.
