# Supplementary figures and images for: Revalidation to single ventricle pathway with single ventricular assist device: Proof of concept
Source: JTCVS Tech. 2024 Mar 7;25:150–2. doi: 10.1016/j.xjtc.2024.02.021 (PMC11184520; doi:10.1016/j.xjtc.2024.02.021)

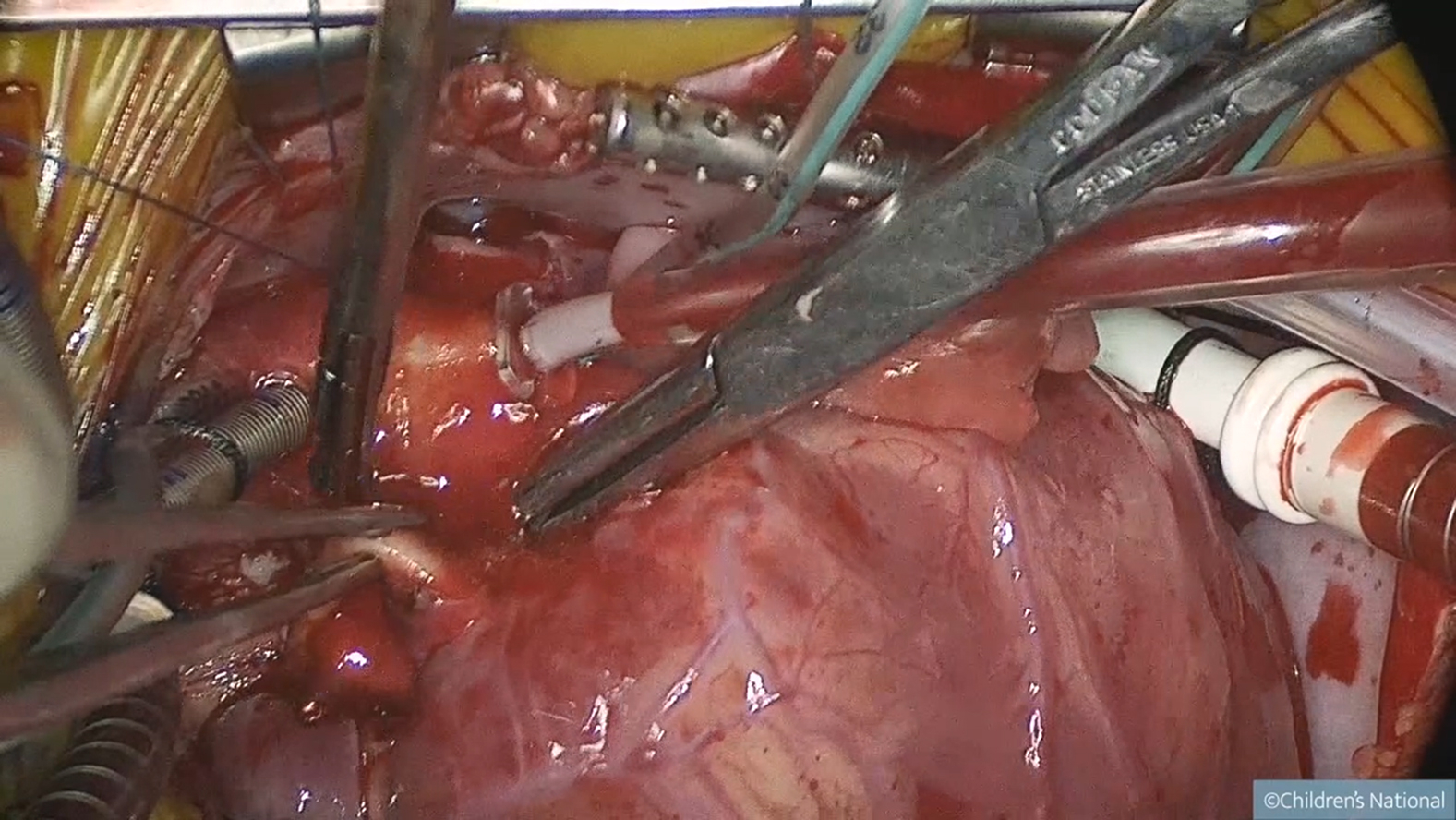

Supplement: Video 1 — Clinical summary of the patient. Video available at: https://www.jtcvs.org/article/S2666-2507(24)00077-4/fulltext. [file fx2.jpg]
